# Incidence of early posterior shoulder dislocation in brachial plexus birth palsy

**DOI:** 10.1186/1749-7221-2-24

**Published:** 2007-12-16

**Authors:** Lars B Dahlin, Kristina Erichs, Charlotte Andersson, Catharina Thornqvist, Clas Backman, Henrik Düppe, Pelle Lindqvist, Marianne Forslund

**Affiliations:** 1 Department of Hand Surgery, Malmö University Hospital, Malmö, Sweden; 2 Child and Habilitation Unit, Malmö University Hospital, Malmö, Sweden; 3 Department of Hand Surgery, Norrland University Hospital, Umeå, Sweden; 4 Department of Orthopaedic Surgery, Malmö University Hospital, Malmö, Sweden; 5 Department of Paediatrics/Urology/Gynecology/Endocrinology, Malmö University Hospital, Malmö, Sweden

## Abstract

**Background:**

Posterior dislocation of the shoulder in brachial plexus birth palsy during the first year of life is rare but the incidence increases with age. The aim was to calculate the incidence of these lesions in children below one year of age.

**Methods:**

The incidence of brachial plexus birth lesion and occurrence of posterior shoulder dislocation was calculated based on a prospective follow up of all brachial plexus patients at an age below one in Malmö municipality, Sweden, 2000–2005.

**Results:**

The incidence of brachial plexus birth palsy was 3.8/1000 living infants and year with a corresponding incidence of posterior shoulder dislocation (history, clinical examination and x-ray) during the first year of 0.28/1000 living infants and year, i.e. 7.3% of all brachial plexus birth palsies.

**Conclusion:**

All children with a brachial plexus birth lesion (incidence 3.8‰) should be screened, above the assessment of neurological recovery, during the first year of life for posterior dislocation of the shoulder (incidence 0.28‰) since such a condition may occur in 7% of children with a brachial plexus birth lesion.

## Background

Brachial plexus birth lesions occur with an incidence of around 2.3–3.3/1000 live births per year [1,2]. Spontaneous recovery is common but as many as 25% of teenagers with a brachial plexus birth lesion may have secondary complications, which are mostly located in the shoulder region with the deformity, medial rotation contracture and problems with activity of daily living (ADL; [3]). An untreated medial rotation contracture may lead to posterior subluxation or dislocation since the natural history of untreated brachial plexus birth palsy with residual weakness is progressive glenohumeral deformity due to persistent muscle imbalance. Progressive deformity has also been found with increasing age [4]. Posterior shoulder dislocation can occur even before the age of one, but the etiology of such an early lesion, which include particularly birth trauma, use of splint devices or muscle imbalance, is still not clarified [5-9]. Recently, the frequency of the condition was reported in consecutive cases with brachial plexus birth palsy below the age of one [5,6]. As many as 8 (11/134) to 10% of the children may have a posterior shoulder dislocation before their first birthday. However, the incidence of posterior dislocation in relation to brachial plexus birth palsy in children below one year of age has not previously been reported. Our aim was to determine the incidence of posterior dislocation of the shoulder among children with an age below one and the corresponding incidence of brachial plexus birth lesion in Malmö municipality, Sweden, during 2000–2005.

## Methods

All children born at Malmö University hospital and living in Malmö municipality (mean population of Malmö 263 550 during the study period) with signs of brachial plexus birth palsy are referred within days for follow-up to the Unit of Child Habilitation. The children have been followed by the same physiotherapist since 1982 (KE) and by a child neurologist (MF). Similar treatment strategies have been adopted during these years, i.e. prophylactic exercises against contracture [regular oral and written (schematic drawing with instruction of specific exercises to prevent particularly shoulder contracture) instructions to parents], regular follow-up of neurological recovery and observation of any signs of development of medial rotation contracture. The procedures have essentially not changed during the time period. Since 1997 most children, and since 2000 all children, with a brachial plexus birth palsy have also been prospectively examined at regular intervals by a hand surgeon (LD) to judge recovery of the neurological deficit of the brachial plexus lesion and particularly any development of shoulder dysfunction including development of contracture and signs of dislocation. A study of persistent symptoms in teenagers with a brachial plexus birth lesion has previously been published from Malmö [3]. The diagnosis of posterior shoulder dislocation was based on the history (sudden development of impaired external rotation), a clinical examination [impaired passive external rotation, asymmetry of the shoulder with palpable humeral head posteriorly, shortening of the length of the upper arm and asymmetry of skin fold due to telescoping of humerus and axillary asymmetry], conventional x-ray (all cases) and MRI/CT (one case).

## Results

**Figure 1:**
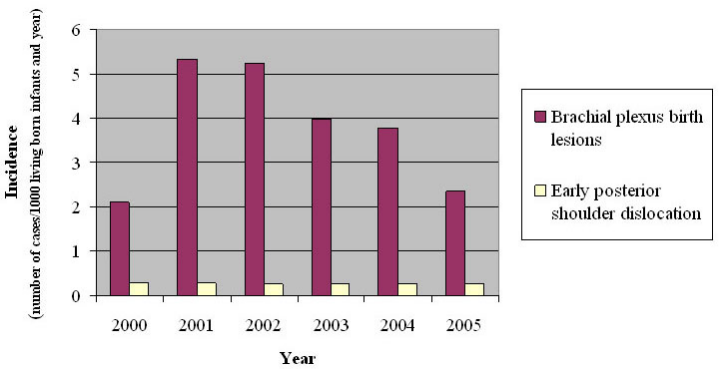
Incidence of brachial plexus birth palsy and early (age less than one year) posterior dislocation of the shoulder per 1000 living born infants and year 2000–2005 in Malmö municipality, Sweden. The numbers correspond to a frequency of 7.3% (six posterior shoulder dislocations out of 82 brachial plexus birth palsies during 2000–2005).

During 2000–2005 21610 living infants were born at Malmö University hospital in Malmö, Sweden. Of these, 82 children had a brachial plexus birth palsy and the children were referred to the Child Habilitation Unit for follow up. The mean incidence of brachial plexus birth palsy was 3.8/1000 living infants per year with a slight variation during the six years (Figure 1). During 2000–2005, we observed one case per year with a posterior shoulder dislocation occurring before the age of 12 months, corresponding to an incidence of posterior shoulder dislocation in such children with brachial plexus birth palsy of 0.28/1000 living children and year, i.e. the frequency of 6/82 (7.3%).

The median birth weight of the six children was 4760 gram (min-max 4100–5340; two boys and four girls). Shoulder dystocia was reported in all six cases. The brachial plexus birth lesion was classified according to Narakas [one case group one (C5–C6), four cases group two (C5, C6, C7) and one case group three (total lesion without Horner)]. In five of the cases there was a spontaneous, not always complete, recovery of neurological function that did not require nerve reconstruction, while the child with the total injury had surgical reconstruction. In that child the C5 and C6 roots were reconstructed with nerve grafts at the age of four months. Posterior shoulder dislocation in the infants was observed in the children before or at the age of 12 months (median 6.5 months; range 4–12 months). No concomitant trauma to the upper extremities was reported among the children except an undislocated humerus fracture at the contralateral side in one girl. All patients, except one (parents declined treatment), were operated on to reposition the humeral head at a mean age of 8 months (range 4–12 months), usually via an anterior exploration with reposition, resection of ligaments, shortening of coracoid process and lengthening of the suprascapular muscle (and conjoined tendon). In the case with total injury botulinum toxin (Botox^®^) was injected into the latissimus dorsi muscle peroperatively. In two patients, additional procedures were done due to the rotation of the humeral head (rotation osteotomy) or recurrence of the dislocation (further anterior release, subscapular release and humerus rotation osteotomy). In all cases (except the child where treatment was denied) the humeral head was correctly located at follow-up [mean follow-up 42 months (2–51; one patient moved after two months)].

## Discussion

In the present paper we describe six of 82 patients with brachial plexus birth palsy who developed a posterior shoulder dislocation during the first year of life. The incidence of a brachial plexus birth lesion was found to be 3.8/1000 live births/year during the study period 2000–2005, with a corresponding incidence of a posterior shoulder dislocation of 0.28/1000 live births per year. The incidence of brachial plexus birth palsy has been reported in previous studies. It has been found to be increased in the western world and various factors related to the occurrence of the lesion have been defined [1,2,10]. Our incidence of brachial plexus birth lesions is somewhat higher than previously reported. We have no clear explanation for this but it may be explained by the fact that since 2000 we see all patients where there is a suspicion of brachial plexus birth lesion (prospective follow up). Thus, we may include in the calculation also patients who recover very rapidly. A posterior shoulder dislocation in children with a brachial plexus birth lesion is known to occur, but is considered to be rare before the age of one. The incidence of a posterior shoulder dislocation before that age has not previously been reported. However, it may occur in as much as 8–10% of the children with a brachial plexus birth palsy before the age of one [5,6,11], which is in accordance with our results (7.3%).

The diagnosis of posterior shoulder dislocation among our six cases was done by the history from the parents and by clinical and radiological examinations (plain x-ray). Unfortunately, ultrasonography of the shoulder [12] was not available at our hospital during the study period. MRI may show deformities of the glenoid in as many as 9 out of 16 children during the first year of age [13]. We did MRI in only one case. The reason was limited MRI resources and the need for anaesthesia during the procedure as previously pointed out by others [7,9]. MRI provides important information about glenoid and articular surface. In the present study, our aim was to confirm the posterior dislocation of the shoulder before surgery thereby not causing any delay for surgery by waiting for an MRI.

Five of the children were operated on to reposition the humeral head, usually with an anterior release and lengthening of subscapular muscle. Recently, arthroscopic release has been reported with successful results even at an age below one [8]. In two of our cases a rotation osteotomy of the humerus was done later while in our third case with dislocation it was more obvious that there was rotation of the humeral head after reposition of the head. In that case an osteotomy of the humerus was done primarily. We suggest that at time of reposition one should consider that a retroversion of the head of the humerus is present [11,14]. Such a condition can be treated immediately at time of reposition with humeral rotation osteotomy [11,15] in order to avoid a second procedure with additional anaesthesia, even if it is more surgically demanding performing the osteotomy at the same time as the release/subscapular lengthening according to the technique by Birch [11]. The advantage being that, one will avoid the possibility of the child being unable to rotate the shoulder medially, a complication described as "play with the hands on the affected side".

Previously, we have not observed early posterior dislocations in infants (< 1 year), although we have followed neurological recovery and shoulder function regularly over the years using the same treatment strategies. We used children followed from 1997–1999 as retrospective controls but we did not find any early dislocations among those children. However, we cannot be sure that we screened all children during that time period. One can not rule out the possibility that the observed number of cases may be explained by our detailed observations, increased awareness, and improved registration of the children thereby finding six cases during the last six years.

Posterior dislocation of the shoulder in connection with brachial plexus birth lesion has been known for 100 years [16-20]. The exact mechanisms by which the condition develops are still incompletely known, but have been discussed in several papers (see for example [5,6,8]). We have used the same treatment strategy, and patient/parents education, to avoid medial rotation contracture. Still we report six cases with posterior shoulder dislocation before the age of one during 2000–2005. Have any procedures changed during the years regarding infants and children that may explain occurrence of posterior shoulder dislocation? Weight bearing on the affected arm during crawling may increase the force of subluxation [5], but may not simply explain the phenomenon. Recommendations to parents regarding the sleeping position of their infants have changed due to the increased risk for sudden infant death syndrome (SIDS; [21]). Among the recommendations to avoid SIDS the parents are advised to let the infant sleep in supine position. During the 1990's a decrease in prone sleeping (decreasing from 32% to 7%) was seen in Sweden in favour of supine sleeping position (increase from 35% to 44%; [21]). Prone sleeping is actually the optimal position for prevention of medial contracture in infants since a passive external rotation of the shoulder (with an abducted shoulder) is stressed in that position during sleep. One may hypothesise that the crucial changes in the sleeping position to avoid SIDS may possibly increase the risk for posterior shoulder dislocation in infants with a brachial plexus birth palsy.

## Conclusion

The incidence of posterior dislocation of the shoulder among children below the age of one with a brachial plexus birth lesion is 0.28/1000 living infants and year (7.3% of all brachial plexus birth lesions). Parents are carefully advised to perform exercises aimed to avoid medial contracture and thereby a posterior dislocation, although early MRI studies observe deformation of the glenoid [13]. We recommend that all children with a brachial plexus birth palsy should regularly, particularly during the first year of life, be examined, not only for extent of neurological recovery, but also with the purpose to early detect a posterior shoulder dislocation.

## Competing interests

The author(s) declare that they have no competing interests.

## Authors' contributions

All authors contributed equally to the article.
